# Damage Control Surgery after Burn Injury: A Narrative Review

**DOI:** 10.3390/ebj3020024

**Published:** 2022-04-01

**Authors:** Hans-Oliver Rennekampff, Mayer Tenenhaus

**Affiliations:** 1 Department of Plastic Surgery, Hand and Burn Surgery, Rhein Maas Klinikum, 52146 Wuerselen, Germany; 2 Independent Researcher, San Diego, CA 92107, USA; m.tenenhaus@sbcglobal.net

**Keywords:** burn, damage control, debridement, surgery, second hit

## Abstract

Burn injuries with cutaneous loss result in a severe systemic response when profound injuries exceed 20% of the total body surface area. The management of severely burned patients is a complex and dynamic process. Timely and safe operative interventions are critical components of multidisciplinary care. Effective management of severely burned patients, their cutaneous injuries, and the associated systemic disease requires a comprehensive understanding of the pathophysiologic response to trauma, objective indicators of patient status, and an appreciation for the dynamic nature of these parameters. Progress in both clinical and basic science research has advanced our understanding of these concepts and our approach to the management of burn patients. Incorporating concepts such as early total care, damage control surgery (DCS), and safe definitive surgery (SDS) in the polytraumatized patient may further aid in optimizing outcomes and quality of care for burn patients. This article connects current knowledge of the lethal triad, inflammation, immunosuppression, and eschar-derived toxins, with surgical burn care, especially burn wound debridement. The concepts of DCS and SDS for the care and management of burn patients are strongly advocated. Experimental and clinical studies are encouraged to validate these concepts in an effort to optimize patient outcomes.

## 1. Introduction

When burn injuries exceed 20% of the total body surface area, the cutaneous insult is further complicated by a severe systemic response. When the thermal insult results in deep partial-thickness or full-thickness damage, surgical excision and skin grafting are generally required. Surgical and intensive care management of the severely burned patient is both complex and dynamic, requiring a comprehensive understanding of the pathophysiology of the injury, the resultant systemic response, and the contributory impact of requisite surgical interventions. While there have certainly been great efforts made in the fields of clinical and basic research as they relate to thermal trauma, tragically, morbidity and mortality remain devastatingly high [1,2]. This sequela is particularly noted in severely burned patients of extremes of age, especially if they have suffered concomitant inhalation injury [1,2]. The establishment of clear algorithms to guide evolving care efforts is needed if we are to further optimize standards and quality of care for our patients, reducing morbidity and mortality. Rotondo et al. [3] first termed damage control surgery (DCS) in 1993, reflecting evolving concepts in resuscitation in an effort to stabilize and reverse the physiologic insult in advance of reconstructive efforts. Since that time, and as a result of its broad adoption, DCS has proven to be one of the most important recent advances in the fields of orthopaedic and abdominal trauma surgery [4,5,6]. Damage control surgery has reduced morbidity and mortality in patients with major trauma and has subsequently been implemented for orthopaedic [4,5], abdominal [6,7], vascular [8], and thoracic injuries [9]. The objective of the damage control strategy is to rapidly control life-threatening injuries and reverse pathophysiological alterations, such as acidosis, hypothermia, and coagulopathy, oft-termed the triad of death [10]. A three-step process was defined that included an abbreviated initial surgery for bleeding and contamination control, aggressive resuscitation in the intensive care unit (ICU), and finally, deferred definitive repair. Key aspects of the triad of death have similarly all been identified in severely burned patients [11,12]. It seems reasonable, then, that both the therapeutic objectives as well as consequences can and should be extrapolated to the management of burn injury patients.

Deciding between a course of damage control surgery and that of early total care remains a particularly challenging dilemma for the burn care specialist. Large wound surface area involvement and inhalation injury are both well-established determinant factors in burn injuries. Judiciously applied high-volume resuscitation remains a critical component in the management of profound burn injuries. Aggressive early debridement and skin grafting with concomitant blood loss can prove life-saving in some and yet detrimental in the compromised patient.

In the following sections, we will introduce and review established as well as newly described physiologic measures that we feel are promising and may ultimately lead to further defining and optimizing care for this devastatingly injured patient population. This narrative review will summarize our current understanding of burn pathology and systemic expression with regard to surgical indications and procedures performed on burn patients.

## 2. Rationale for Use of Damage Control Surgery in Burn Patients

It is critical to appreciate that while burn injury patients share many physiologic and interventional commonalities with other forms or presentations of major traumatic injuries, burn injury patients manifest a unique set of pathophysiologic insults. This appreciation helped justify and promote the establishment of specialized burn units.

Many burn centres currently employ and rely upon individually defined protocols, which are derived from current and established guidelines, tempered by experience, training, practice, and logistical considerations. These protocols allow for the uniform and efficient translation of care amongst personnel, in an effort to efficiently and methodically guide care.

In an effort to further diminish morbidity and mortality rates among this unique patient population, it is imperative to introduce new data and developments in re-examining and re-establishing treatment protocols and guidelines, as well as the critical timing of therapeutic interventions. Lessons learned from evolving orthopaedic and surgical trauma efforts may similarly prove beneficial for burn patients. It is only through future objective study that we can improve our patients’ survival and subsequent quality of life.

Damage control surgery complements this practice and could potentially further improve outcomes. There is, however, only spare evidence as to which burn patients would benefit from deferred early total care and gain from damage control surgery. Discussions of what exactly constitutes early excisional approaches continue to be argued and challenged.

Hypothermia has been identified as a major responsible factor for mortality in burned patients: Muthukumar et al. [12] identified a 4.27-fold risk of death in patients presenting with a mean hypothermia of 34.8 °C, and Hostler et al. [13] reported that hypothermia was independently associated with mortality. Ziegler et al. [14] described a positive influence of normothermia (≥36 °C) on survival. In contrast, Sherren et al. [11] reported that the lethal triad in burn patients was associated with the severity of the injury as measured by the Abbreviated Burn Severity Index (ABSI) but did not reflect the patients’ outcomes. Similarly, Ehrl et al. [15] could not identify a significant difference in mortality between normothermic and hypothermic patients; however, time until death was shorter in hypothermic patients. Regarding intraoperative hypothermia, an increased risk of infectious complications by a rate of 1.4 was reported [16]. In summary, hypothermia seems to be a critical parameter that correlates with morbidity and mortality. Thus, hypothermic patients may benefit from the concept of damage control surgery in combination with warming measures, rather than early total care [17].

The second factor of the lethal triad, namely coagulopathy, is certainly reported and appreciated in thermally injured patients [11,18]. Fibrinolytic dysfunction has been identified as early as 1 to 2 hrs post insult. Garcia-Avello et al. [19] reported that burn patients exhibited hypercoagulability and hyperfibrinolysis beginning as early as post-burn day 1 and persisted until post-burn day 7. This derangement of coagulation factors was more pronounced in non-survivors and appears to be related to organ failure. Pusateri et al. [20] and Sherren et al. [21] reported that early fibrinolytic dysfunction as measured by thromboelastography was associated with increased mortality in burn patients. Huzar et al. [22] noted that hypocoagulability was negatively associated with mortality. Taken in concert, these reports demonstrate that analysing coagulation parameters in burn patients may identify patients at risk for deleterious outcomes.

Acidaemia has also been shown to correlate with a poorer outcome in severe burn injury [23,24], thereby establishing all three factors of the lethal triad in severe burn injury.

Orthopaedic traumatologists have increasingly recognized the importance of soft tissue injury in overall patient morbidity and mortality. As a result, injury scoring measures have added soft tissue injury as a fourth parameter when assessing orthopaedic trauma patients who have suffered concomitant injuries to extremities, lungs, the abdomen, and the pelvis [4,25,26,27]. Cutaneous soft tissue loss is a hallmark of burn injury and has been identified as an independent risk factor in burn lethality [28,29]. While fundamental questions as to the optimal time to perform debridement and even definitive skin grafting persist, burn patients with critical findings of the lethal triad and extensive TBSA burns may well benefit from DC surgery, and this in turn may improve survival.

Pape et al. [26] described four classes of orthopaedic trauma patients, according distinct parameters from the following categories: shock, coagulation, temperature, and soft tissue injury, as either “stable”, “borderline”, “unstable”, or “in extremis” (see Table 3 in [26]). The authors recognized that immediate surgery would significantly and negatively impact patients in the latter two groups, leading to organ failure or even death. “Borderline” patients who are initially stable may worsen after surgery [26]. In an effort to better define this subset, several negative prognostic factors have been described for “borderliners”. To date, similar clear or identifying characterizations for burn patients at risk have not been fully established and described; however, detrimental prognostic factors, such as inhalation injury and the total body surface area of burned skin, are well-recognized for burn patients, which, in like manner, justify pursuing a DC surgical approach.

To achieve the goals of both timely debridement and safe grafting in severely burned patients, a precise knowledge about the patient’s status seems crucial. Several scoring systems on mortality or survivability have been reported for burn patients. Most of these burn injury-related scores (Baux score, revised Baux score, Abbreviated Burn Severity Index (ABSI) score, Ryan score, and Belgium Outcome Burn Injury (BOBI) score) are based on total body surface area (TBSA), age, and inhalational trauma (IHT) [28,29], but do not specifically respect pathophysiological data of the lethal triad [30]. Currently, none of the prognostic burn scores are utilized for strategic planning of surgical procedures in burn patients.

## 3. Second Hit and Early Neutrophil Changes in Burn Patients and Their Prognostic Relevance

It is now well-accepted that an initial trauma (e.g., burn injury) leads to a systemic inflammatory response, which can be exaggerated by a secondary stimulus (e.g., infection, endotoxin, or additional surgery) [30]. This phenomenon has been termed the “two-hit” hypothesis [31]. A variety of cell types, such as macrophages and polymorphonuclear leukocytes (PMNs), are involved in this staged response [32,33]. Although PMNs are critically important for host defence, excessive activation and their accumulation in organs, such as the lung, with subsequent release of free oxygen radicals and mediators, can lead to tissue injury. These effects may manifest locally, distantly, and systemically. Interestingly, both hyper- as well as hypoactivity of PMNs have been linked to the initial systemic inflammatory response syndrome (SIRS), followed by the compensatory anti-inflammatory syndrome (CARS). The early overwhelming inflammatory response and the secondary occurring immunoparalysis are considered key factors for the development of secondary infection resulting in the high morbidity and mortality rates observed in severely burned patients. Previously, specific soluble factors occurring post trauma (alarmins and damage-associated molecular pattern (DAMP)) have been analysed and linked to initial inflammation, activation, recruitment of PMNs, and altered immune function [34,35,36]. While the expression of DAMPs post burn has been analysed in detail and a therapeutic approach suggested [37,38], knowledge of DAMP expression has not been used for the strategic planning of safe surgical procedures. Secondary to DAMP activation of PMNs, a profound cytokine release has been identified. Utilizing proteomics, Finnerty et al. identified a set of proteins that were differently expressed in non-survivors as compared to survivors with a similar total body surface area (TBSA) of third-degree burns [39]. The Glue Grant Project has further identified burn injury-induced inflammatory and immunological changes by genetic profiling. In burns exceeding 20% of the TBSA, leukocyte gene expression was modestly altered, while burns exceeding 40% of the TBSA resulted in a significant dysregulation of several genes (genomic storm) in leucocytes [40]. It is now generally accepted that severe burn injuries alter innate immunity with compensatory anti-inflammatory responses, together with downregulation of adaptive immunity [41,42,43]. Regarding the timing of burn surgery, we hypothesize that PMN-related immunological changes should be taken into account if one is to minimize complication rates [44].

Identifying safe and optimal “windows of opportunity” for definitive intervention is critical if we are to establish useful, judicious, and responsible guidelines. Orthopaedic trauma surgeons [45] recognize post-trauma days 2 to 4 as unsuitable for performing definitive osteosynthesis, while identifying post-trauma days 5 through 8 as a “window of opportunity” for definitive and reliable intervention. In burn surgery, early debridement within 3 to 5 days has often been advocated, although the exact timing (e.g., <24 h or < 72 h) of early escharotomy remains somewhat of a matter of debate. A waiting period with antimicrobial conservative management and late debridement was reported to increase LOS and infectious complications [46,47]. The conflicting data on the effect of timing of burn surgery on outcomes ask for reliable parameters to identify patients at risk for complications after extensive early surgery.

Analysis of cytokine profiles, white blood cell ratio, and the measurement of subsets of neutrophils have been advocated as markers for the severity of burn injuries and predicting death [48,49,50]. While point-of-care measurements of neutrophil activation in trauma patients has become available [51], altered neutrophil function guiding burn surgery has to date not been definitively established. After major burn trauma, a number of important cell surface receptors on PMNs are altered [50,52,53]. Toll-like receptor 4 (TLR4) is expressed on PMNs, and lipopolysaccharide (LPS) as well as DAMPs are recognized as activators of TLR4. TLR4, in collaboration with other Toll-like receptors such as TLR2, recognize LPSs of Gram-negative bacteria, inducing septic shock. Integrins, which are transmembrane receptors, mediate cellular signals and facilitate rapid responses to events at the cell surface. CD11b is one of the well-established activation markers governing PMN extravasation and superoxide release. A series of reports described alterations in CD11b expression on PMNs in burn patients, with conflicting data regarding the level of expression [50,52,53]. A correlation of CD11b expression in full-thickness burns, but not total body surface area (TBSA), was observed, while CD16 expression on PMNs did correlate with TBSA burns [50]. Recent publications from the orthopaedic trauma literature describe a subset of PMNs (CD16dim/CD62Lbright) that correlated with severity of injury [51,54]. Further quantitative analysis of this cell type allowed for predicting infectious complications. In the future, this interesting finding may allow for the identification of patients at particular risk for infectious complications after suffering severe trauma. We advocate similar point-of-care measurements for burn patients in an effort to plan safe surgery.

Researchers have also focused on the functionality of PMNs, describing a generally decreased oxidative response to potent mediators for inflammatory reactions, such as the N-formyl-Met-Leu-Phe protein (fMLP) in severe traumatic injury and a significantly decreased response in patients developing complications [55,56,57]. In contrast, the group from the Linköping burn centre [43] could not identify a correlation with burned area or area of full-thickness burns and respiratory burst activity. If the percentage of TBSA burns and full-thickness burn area have no influence on the observed decreased respiratory burst of PMNs, current scores including TBSA and full-thickness burns will not be able to comprehensively identify patients at risk for major complications due to PMN hypofunction.

## 4. Burn Toxins and Debridement of the Burn Eschar—What Is the Clinical Evidence?

Our innate immune system has the capacity to detect and respond to a variety of critical stimuli, such as “non-self” molecules derived from pathogens and other insults. Burn eschar, a reservoir of damaged and dying cells, releases endogenous host-derived molecules termed damage-associated molecular patterns (DAMPs), which bind to receptors expressed on innate immune cells, notably Toll-like receptors (TLRs), promoting an inflammatory response [58,59,60]. In cases of significant burn injury, the collective DAMP-associated response, as introduced above, results in a cascade of deleterious systemic effects, specifically a dysregulated sterile inflammatory state, which when severe, culminates in a profound state of immunosuppression. In the last century, Allgöwer et al. [61,62] identified burn tissue-derived toxins responsible for severe immunosuppression and mortality. An elegant set of experiments by Hansbrough et al. [63] demonstrated that burn tissue was at least one causal source for the detrimental immunological effects observed in burn injuries. These clinicians and others strongly advocated for the elimination of burn toxins from the skin, either by primary excision of the burns [64,65,66] or local application of a protein-complex-binding substance such as cerium nitrate [67,68,69,70]. In concert with this direction, clinical evidence has repeatedly demonstrated that patients with extensive burns (>30% of the TBSA), whose eschars were excised early, showed reductions in circulating cytokines, hypermetabolism, and mortality. Data derived from a variety of early versus late (> day 6) eschar removals performed in animal studies concluded that early debridement prevented the often-seen profound inflammatory state and immunologic dysfunction associated with such injuries, as well as promoting an accelerated re-epithelialization and reduction in scarring. A 2006 meta-analysis [46] reviewed prospective studies comparing early excision and grafting with delayed grafting after eschar separation (a historical approach) in burn patients without inhalation injury. In this meta-analysis, survival was improved in the early excision group, with a shortened length of stay (LOS). Of particular note, early excision was reported as spanning a period of as early as < 24 hrs post burn to the 6th post-burn day, making a recommendation on exact timing difficult. In contrast, a very recent review on regrouped data [71] described that mortality was lower in late excision (>7 days) compared to early excision (<6 days), while early excision reduced septic episodes and LOS. In an experimental burn model, we were able to demonstrate that very early debridement of the burn wound led to an exaggerated sequestration of polymorphonuclear leucocytes (PMNs) in the lung [72]. The impact or “second hit” of surgical debridement may explain the subsequent cascade of effects resulting from early excision of the burn eschar. In line with this assumption, data from the German Burn Registry [73] confirmed the negative impact of full-thickness burns on survival, possibly indicating that the impact of surgical intervention may have a negative effect on outcomes. These results underscore the relevance and importance of a second hit phenomenon and ask for an individualized damage control surgical approach in order to reduce DAMPs and improve outcomes.

Allogenic blood transfusion is well-recognized as an independent factor for immunosuppression in trauma patients [74,75]. It is extremely important to appreciate that in the aforementioned meta-analysis [46], early excision was accompanied by increase blood transfusion requirements. In order to achieve a timely debridement with reduced blood transfusion requirements, enzymatic debridement has been advocated [76,77]. Several forms of enzymatic debriding agents have been developed and promoted, expressing differing potencies and efficiencies. At least one randomized controlled clinical study has demonstrated that enzymatic debridement of up to 15% of the TBSA can safely be performed, accompanied by a reduced blood transfusion rate in the enzymatically debrided group as compared to the conventional surgically debrided burn patients [78,79]. It is hypothesized that in severely burned patients, this evolving technique may reduce the second hit. At present, further study and proof is needed; still, timely debridement remains the clinical standard for full-thickness burns.

The burn eschar is at considerable risk of becoming a nidus for pathogenic bacterial and fungal growth, with subsequent systemic complications such as sepsis, particularly as tissue and cellular integrity and vitality are compromised. Further complicating the clinical challenge of caring for severely injured patients, and particularly burn injury patients, is the contribution of their premorbid and morbid medical status, which may not always allow for safe, early surgical debridement. A very recent report described the binding of DAMPs from eschar by topical cerium nitrate, leading to a reduced systemic inflammatory response and modulating the over-activation of PMNs and immunologic dysfunction [37]. Interestingly, as early as 1976 [80], topically applied cerium nitrate, a potent antiseptic, was clinically reported to reduce mortality in burn patients. Dutch burn surgeons have shown that the introduction of topical cerium nitrate may allow for safe serial surgical debridement on the compromised burn patient [69]. Further studies will have to show the precise role of topical cerium nitrate in damage control burn treatment.

## 5. Transfusion Requirements and Control of Blood Loss in Burn Patients

As we touched upon earlier in this text, significant blood loss, upwards of 200 mL/% TBSA, can and does occur during surgical debridement. This often results in the need for considerable volumes of blood and blood-derived component transfusions [81,82,83]. Several studies have demonstrated that patients who have suffered burn injuries of greater than 20% of TBSA received at least one red blood cell (RBC) transfusion during their hospital stay [84]. It is imperative that reliable guidelines are enacted to optimize safety and efficacy while reflecting currently evolving standards of care. Transfusion requirements must be individualized to account for a patient’s specific needs and reserve, be it cardiopulmonary reserve, inherent or reactive coagulopathies, nutritional, or other such premorbid and morbid considerations [85]. Ultimately, the patient’s clinical status must guide the necessity for RBC transfusions. In this regard, the clinical adequacy of oxygen delivery continues to prove particularly difficult to objectively assess, and as such, we are left with calculations of mixed venous O_2_ levels and O_2_ extraction ratios while factoring in the anticipated degree and rate of blood loss, the effect of body temperature, and medications, such as anaesthetics, on oxygen consumption. The American Society of Anesthesiologists Task Force (ASATF) recommends that RBCs should usually be administered when the haemoglobin concentration is low (for example, <6 g/dL in a young healthy patient), especially when the anaemia is acute [86]. Furthermore, the ASATF states that RBCs are usually unnecessary when the Hgb concentration is >10 g/dL. These guidelines may and should be qualified in cases of anticipated blood loss. This is the purview of the burn and trauma care team. The adverse effects of large volume blood transfusions include immunosuppression, which may result in an increased risk of infection, a predominant cause of death in burn patients. Transfusion-related acute lung injury (TRALI), SIRS, sepsis, thrombotic complications, electrolyte and dilutional challenges, hypothermia, transfusion-associated graft-vs-host disease (TA-GVHD), and many other potentially life-threatening complications [87] have all been causally related to large volume blood and blood product transfusions. Transfusion requirements may well account for a second hit adversely effecting final outcomes.

From a clinical perspective, ascertaining haemoglobin levels during surgery and bleeding remains lacking, as it imprecisely measures a critical determinant, specifically that of tissue oxygenation. Restricted transfusion regimens, evolving management of intraoperative coagulopathy, warming, and other interoperative adjunctive techniques are certainly advocated in an effort to further reduce transfusion rates and optimize outcomes. Of particular interest was a large prospective multicentre clinical trial (TRIBE) on restricted blood transfusion in burn patients [84]. Two principle groups were compared. The first group was a control group, which had a transfusion trigger of <10 g/dL and received a median of 16 RBCs. The second group, the patient group (median TBSA: 31%, median number of operations: two, and median LOS: 31 days), received restrictive transfusion management for a transfusion trigger of <7 g/dL, accounting for a median transfusion rate of eight units of RBCs during their stay. A total of 16% of this latter group received no transfusion at all. This study did not demonstrate a reduction in blood stream infection, wound infection, mortality, or length of stay [84]. It appears then that even a 50% reduction in transfusion requirements may not be sufficient to reduce the impact of blood transfusions. In a review by Dr. T. Palmieri [88], she concluded that “fresh-frozen plasma and platelets during burn excision of more than 20% may decrease transfusion requirements”. Many authors [81,89,90] have stated that RBC transfusion is not, in and of itself, haemostatic and asked for a stronger focus on intraoperative monitoring of coagulation disorders and related substitution therapy. A subsequent review [91] on the management of bleeding identified haemostatic monitoring by thromboelastographic or rotational thromboelastometry as an improved measure to decrease transfusion rates. In addition to fresh frozen plasma and platelet transfusion, recent articles [92,93,94] have advocated the use of intravenous tranexamic acid, a fibrinolytic inhibitor, to reduce blood loss and transfusion requirements in burn wound excision. Currently employed strategies to reduce blood loss during excisional surgery, in addition to those mentioned above, include tourniquet application when appropriate, tumescent techniques, and the application of topical agents for vasoconstriction and haemostasis, such as thrombin, fibrin sealants, and epinephrine. Similar measures can be employed when creating and treating donor sites during the harvesting of skin grafts and in the preparation of the wound bed for grafting. Applying a restricted blood transfusion policy, correcting intraoperative coagulopathy, and controlling local bleeding by various means will make burn surgery safer and reduce a second impact for the burn patient.

## 6. Future Aspects of Damage Control (DC) Burn Management and Safe Definitive Surgery (SDS) in Burns

Burn injuries alone or in combination with inhalation injury constitute one of the most complex pathophysiologic systemic insults, with dynamic and rapidly evolving effects transpiring over a very short period of time. As a result of this injury pattern and despite tireless efforts and lessons learned, mortality remains high. Effective burn treatment requires a profound knowledge of this rather unique pathophysiology in order to execute safe, timely, and optimal care.

The patient’s status or severity of injury is primarily defined by age and burn size. It is accepted that burn trauma is a “first hit”, with a resultant dramatic inflammatory response accompanied by changes in the innate immune system (e.g., neutrophils). There is evidence that additional surgery, such as that seen with burn wound excision, leads to a “second hit phenomenon”, which may further compromise the burn patient. Excision of the burned tissue followed by skin transplantation remains the standard of care for the treatment of deep burn injuries. This course has definitively been shown to reduce morbidity and mortality. Excising the burn eschar removes dead and dying tissue from local and systemic interaction; the concomitant reduction of DAMPs seems a rational approach. The extent of excision, timing of the procedures, and applied debridement techniques are not generally standardized among burn centres, leaving much room for improvement and unfortunately adverse events.

Enzymatic debridement and topical application of cerium nitrate are just a few examples of techniques currently available that may alter current burn management. Knowledge of which patients might benefit from such strategies unfortunately remains lacking, further bolstering the need for a comprehensive methodology to establish optimal damage control management.

Up to now, several burn-related scores and single soluble parameters have been described to either classify the severity of burns or predict survival. While these efforts have certainly shown promise and aid in care, these scores and combinations of parameters have not been applied to identify patients who may benefit from a broader damage control approach. Orthopaedic trauma surgeons concluded that multiple scores in combination with additional parameters such as lactate clearance must be employed to categorize patients for either safe definitive surgery or a damage control approach. A polytraumatized patient classification system indicating who may benefit from definitive surgery or damage control surgery has been published (see Table 3 in [28]). A consensus among burn surgeons to adapt this classification may prove helpful and, in our estimation, necessary if we are to continue to advance and execute ever safer and effective burn surgery and care. For pure burn injuries, i.e., those who did not suffer additional multitrauma, such as head injury, long bone injury, etc., we propose replacement of the variable “blood units” by “amount of resuscitation volume”, “chest trauma” by “inhalation injury”, and “pelvic fracture classification” by “TBSA” or a burn-related score. Additional advances in technical developments, such as point-of-care flow cytometry (FACS) analysis, may allow for analysis of PMN subsets and their functions, such as respiratory burst capacity. These findings will be helpful in defining patients at risk for extensive or prolonged surgical procedures. While general guidelines exist for identifying and addressing inhalation injury, no clear recommendation is afforded to us as to how and when to execute safe surgery in this complex condition that is so well-known to profoundly and negatively affect survival.

In the future, well-designed prospective studies are warranted to evaluate the benefit of damage control management versus definitive surgery for burn patients. Assessment of patient immune status is needed to more accurately and better categorize and address dynamic burn patient risk profiles, as well as the evolving physiologic state of injury response, as opposed to solely relying on extent of injury (TBSA) and accompanied diseases such as inhalation injury. In our lifetime, we have seen and participated in the development and application of significant improvements in our ability to treat and care for burn patients. This process deserves continued reflection and attention. Damage control resuscitation and point-of-care measurements to direct and correct this incredibly complex pathophysiologic disease demands it. It is likely that establishing more comprehensive guidelines, addressing our rapidly evolving understanding of coagulation pathology and lactate clearance, will further improve the quality and efficacy of our burn care efforts and make burn surgery safer.
